# Successfully intravenous thrombolytic therapy in systemic lupus erythematosus-related ischemic stroke: A case report

**DOI:** 10.1097/MD.0000000000040203

**Published:** 2024-10-25

**Authors:** Guanglin Liu, Yong Wang, Hongjian Guan

**Affiliations:** a Stroke Center, Department of Neurology, Yanbian University Hospital, Yanji, Jilin, China; b Medical College, Yanbian University, Yanji, Jilin, China.

**Keywords:** case report, high-resolution magnetic resonance image, ischemic stroke, systemic lupus erythematosus, thrombus-associated antibodies

## Abstract

**Rationale:**

Stroke is a relatively frequent complication occurring in patients with systemic lupus erythematosus (SLE). The increasing number of patients with Ischemic Stroke secondary to SLE aroused the clinician’s concern. SLE thrombosis markers, diagnostic high-resolution magnetic resonance image (HR-MRI), and therapeutic interventions for acute ischemic stroke were recently coming into focus perspectives from the field.

**Patient concerns:**

A 42-year-old female with slurred speech and numbness in her left limb was admitted to our hospital.

**Diagnoses:**

Magnetic resonance imaging (MRI) revealed right thalamic infarction with diffusion-weighted lesions. Prior to admission, the patient had a National Institute of Health Stroke Scale (NIHSS) score of 3.

**Interventions:**

In light of the clinical manifestation, the American Heart Association/American Stroke Association (AHA/ASA) Guidelines for Intravenous Thrombolysis in Acute Ischemic Stroke (2019) should be referred to. The patient was treated with thrombolytic alteplase (rt-PA).

**Outcomes:**

The patient was hospitalized for 2 weeks and discharged after his symptoms improved.

**Lessons:**

After thrombolysis, the NIHSS score of the patient decreased to zero. The computed tomography scan was reexamined 24 hours later, and no acute changes or hemorrhage were identified in the infarcted area. Subsequent imaging and serological analyses indicated that HR-MRI of the responsible vessel was negative, but the infarction in this patient was still regarded as being caused by vasculitis of the right posterior cerebral artery in the region supplying the thalamus. This is the first case of successful intravenous thrombolytic therapy with rt-PA in a patient with SLE secondary to stroke with an NIHSS score of 3. This provides further evidence for expanding the reference of indications with rt-PA intravenous thrombolysis.

## 1. Introduction

Acute ischemic stroke (AIS) is a complication of SLE and is particularly common in younger patients.^[1]^ The pathogenesis of stroke secondary to SLE is increasingly being linked to Libman–Sacks endocarditis, abnormal blood components, cytotoxic treatment regimens, and rare brain arteritis.^[2]^ However, the diagnosis and treatment of stroke secondary to SLE is still a matter of limited clinical experience and a lack of expert guideline consensus. Currently, only 4 cases^[3–6]^ of SLE-secondary stroke treated with intravenous thrombolysis using rt-PA have been reported, as well as 1 case^[7]^ of SLE-associated stroke in which mechanical thrombectomy was performed. According to the UK National Clinical Guidelines for Stroke, an NHISS score of less than or equal to 3 is used by academics as a criterion for minor ischemic stroke.^[8]^ However, no case of successful intravenous thrombolysis with rt-PA in a patient with SLE secondary to minor ischemic stroke has been reported to date. In this article, we report a 42-year-old female patient with a history of SLE who developed an acute infarction in the blood supply region of the thalamic leading to minor ischemic stroke. Following rt-PA thrombolysis, successful revascularization of the right thalamogeniculate artery (PCA-P2 segment) was achieved. By reviewing the literature on intravenous thrombolysis, mechanical thrombectomy, and pharmacological conservative treatment of stroke secondary to SLE, this article further reviews the diagnostic and therapeutic strategies for stroke secondary to SLE.

## 2. Case report

**Figure 1.**
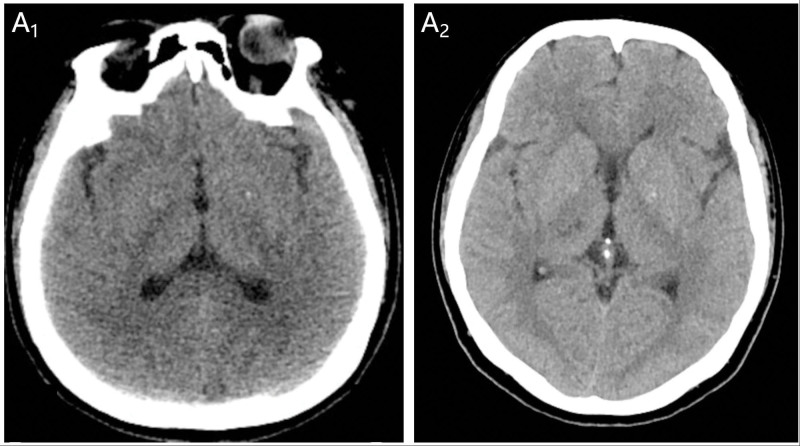
Computed tomography (CT) images during thrombolytic therapy with alteplase. (A_1_) On admission, CT of the head was normal. (A_2_) Twenty-four hours after the end of intravenous thrombolysis, the reexamination of the head CT showed no hemorrhage.

**Figure 2.**
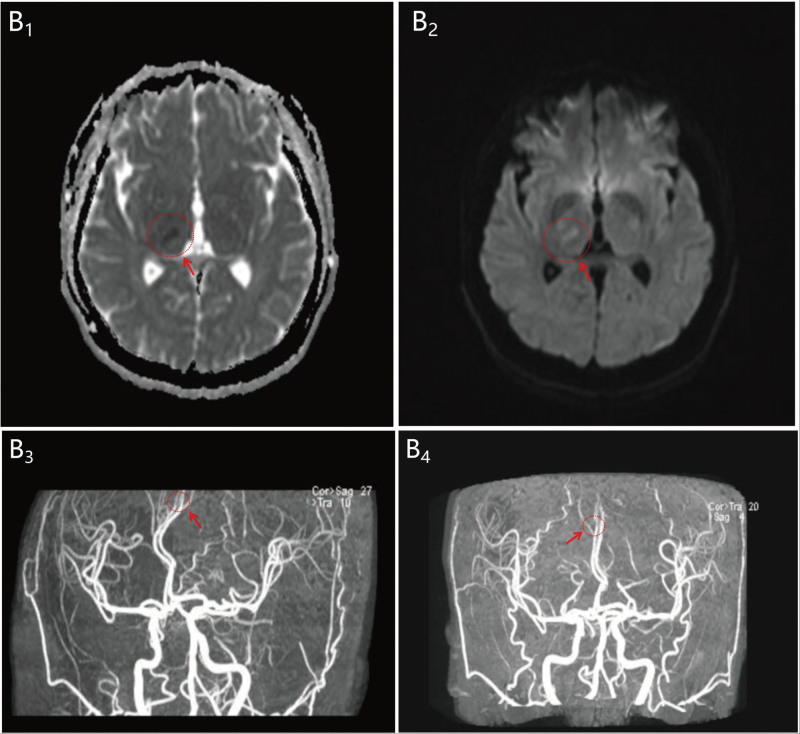
Magnetic Resonance Imaging (MRI). (B_1_ and B_2_) An MRI examination of the head displayed the right thalamus was speckled with abnormal signal, a slightly high signal on DWI, and a low signal on ADC. (B_3_ and B_4_) Intracranial magnetic resonance angiography excluded intracranial large vessel occlusion and showed a right anterior cerebral artery (A4 segment) stenosis.

**Figure 3.**
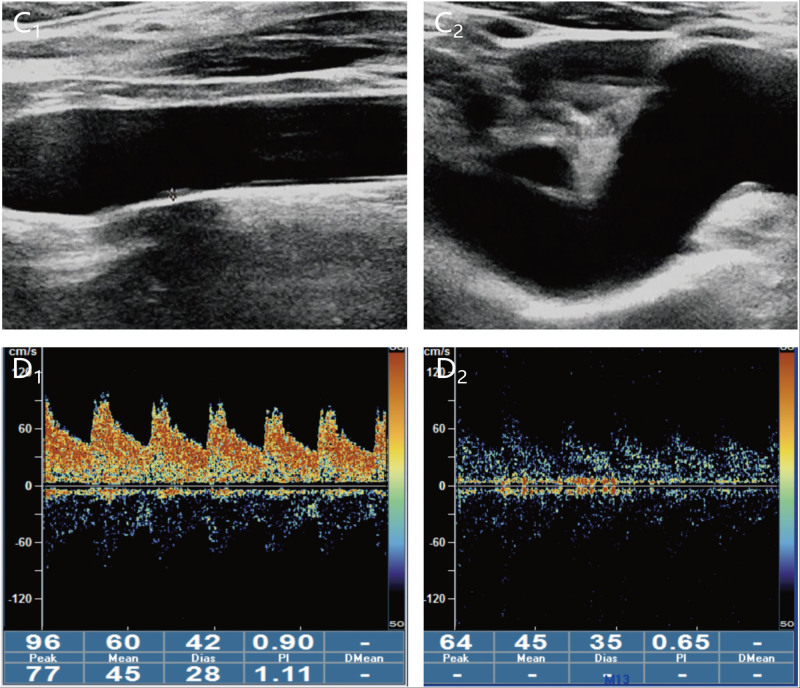
Carotid Ultrasound and Transcranial Doppler bubble Test. (C_1_ and C_2_, D_1_ and D_2_) No obvious abnormality was found in the carotid ultrasound and transcranial Doppler bubble test.

**Figure 4.**
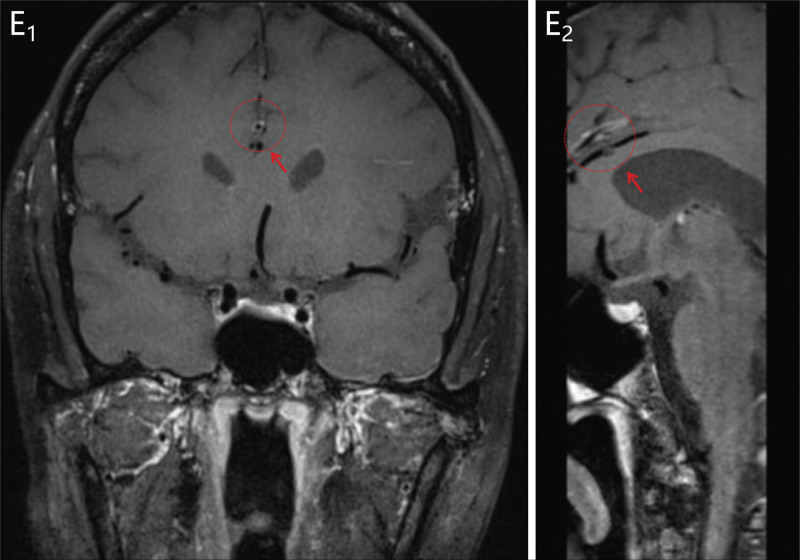
High-resolution Magnetic Resonance Image (HR-MRI). (E_1_ and E_2_) The high-resolution magnetic resonance image (HR-MRI) shows vascular inflammatory changes in the anterior cerebral artery with ring enhancement, suggesting arterial vasculitis. There were no atherosclerotic changes observed in the right posterior cerebral artery.

A 42-year-old female presented to the emergency department of Yanbian University Hospital with numbness of the left limb for 1 hour. On 5th November 2023, the patient noticed that she had developed numbness in the left upper and lower extremities and tightness in the left side of her face. The patient underwent a computed tomography (CT) scan in the emergency department, and no obvious abnormalities were found (Fig. 1). The patient was eligible for rt-PA intravenous thrombolysis within the time window, and contraindications were ruled out. After explaining the benefits and risks of the treatment to the family, they agreed to proceed with the rt-PA intravenous thrombolysis. The DNT (door-to-needle time) was 29 minutes. Immediately after the end of thrombolysis, the patient underwent magnetic resonance angiography and angiography, which indicated that the right thalamus was speckled with an abnormal signal, a slightly high signal on DWI, and a low signal on ADC (Fig. 2). Magnetic resonance angiography excluded intracranial large vessel occlusion and showed a right anterior cerebral artery (A4 segment) stenosis (Fig. 2). She had 3 years of hypertension and was treated with antihypertensive drug treatment (30 mg nifedipine controlled-release tablets), but her blood pressure was still unstable. The patient had a history of SLE and was treated with a combination of methylprednisolone and hydroxychloroquine. Regular follow-up appointments were conducted at the Department of Immunology of Yanbian University Hospital. The patient also had a history of 1 cesarean section and 1 abortion. Physical examination revealed a body temperature of 36.3 °C, pulse rate of 96 beats/min, blood pressure of 189/115 mm Hg, and respiratory rate of 20/min. The patient presented with scattered nodular erythema and brown pigmentation on both lower limbs. She presented with pain at the small joints of the hand [i.e., metacarpophalangeal (MCP) and proximal interphalangeal (PIP) joints] and wrists joints and knee joints. The neurological examination revealed grade 4 muscle strength in the left limbs, normal muscle tone, and hypesthesia on the left side. National Institute of Health stroke scale (NIHSS) score of 3 (1 point for dysarthria 1 point for facial palsy and 1 point for hypesthesia). One hour after the onset of symptoms, intravenous thrombolysis (intravenous rt-PA) was initiated at a standardized dose of 0.9 mg/kg,^[9]^ resulting in a total dose of 55.8 mg. The initial injection consisted of 5.58 mg (10% of the total dose), followed by the infusion of the remaining dose over 1 hour. During the thrombolysis procedure, the patient developed a headache, leading to the discontinuation of the treatment. Following this, the patient received a head CT examination, which did not reveal any signs of intracranial hemorrhage, thus enabling the resumption of thrombolysis. The symptom resolution of the patient was significant and her NIHSS score 1 hour after thrombolysis was 0 points. About 24 hours after receiving intravenous thrombolysis, the patient underwent a CT scan of the head, which revealed patchy hypo-density in the area of the right thalamus and basal ganglia (Fig. 1). The cerebrovascular event of the patient appears to have occurred during an active phase of SLE disease, as suggested by their SLE blood marker tests and follow-up results (Table 1). No significant abnormal results were found in carotid artery ultrasonography, vertebral artery ultrasonography, and contrast transcranial Doppler (Fig. 3). The HR-MRI shows vascular inflammatory changes in the anterior cerebral artery with ring enhancement, suggesting arterial vasculitis. There were no atherosclerotic changes observed in the right posterior cerebral artery, and plaque was considered (Fig. 4). The patient was discharged from the hospital on November 22, 2023. After discharge from the hospital, the patient received secondary prevention in ischemic stroke patients and subsequent further treatment with Belimumab in the immunology department. Following the follow-up, it was revealed that the patient did not suffer from any new strokes within 30 days of being discharged. To prevent further cerebral arterial inflammation caused by SLE, the patient underwent regular follow-up care at the Department of Immunology, Yanbian University Hospital.

**Table 1:** Changes in biomarkers of SLE.

Biomarker	Date				Unit	Reference value
	March 31, 2021	July 8, 2021	August 12, 2022	November 8, 2023		
ANA	1:1000	1:1000	–	1:3200	–	<1:100
Sm	2.08	3.16	–	<2.00	RU/mL	0 to 20
nRNP/Sm	6.29	<2.00	–	<2.00	RU/mL	0 to 20
dsDNA	28.34	4.65	–	3.75	IU/mL	0 to 10
C3	0.49	0.71	0.59	0.70	g/L	0.1 to 0.4
C4	0.06	0.11	0.13	0.13	g/L	0.9 to 1.8
IgA	2.32	1.65	1.81	1.82	g/L	0.7 to 4.0
IgM	0.39	0.66	0.47	0.49	g/L	0.4 to 2.3
IgG	20.50	13.30	18.0	18.2	g/L	7 to 16

## 3. Literature review and discussion

### 3.1. The current state of epidemiology

In recent years, a growing number of researchers have shifted their focus to the area of acute ischemic stroke secondary to SLE. A comprehensive cohort study conducted in Taiwan revealed a striking 3.2-fold increase in the risk of stroke among the SLE cohort compared to their counterparts without SLE.^[10]^ The incidence of acute ischemic stroke secondary to SLE is about 2.4, 4.5, and 32.9 per 100,000 persons in the age groups of 20 to 24, 30 to 34, and 45 to 49 years, respectively.^[11]^ A recent study indicated an increased risk of stroke in SLE patients during the first year of SLE diagnosis and the use of steroid medications.^[12]^ Furthermore, hypertension and kidney disease play crucial roles in predicting the onset of stroke in the SLE population. This evidence suggests that factors secondary to stroke in patients with SLE may be associated with increased disease activity in SLE.

## 4. Pathogenic mechanisms

There may be several pathogenetic mechanisms for stroke secondary to SLE. These include cardiogenic embolism, atherosclerosis or non-atherosclerotic stenosis, abnormal blood composition, cytotoxic therapeutic regimens, a rare inflammation of cerebral arteries, and aseptic infections.^[13,14]^ Moyssakis et al reported that around 30% of SLE patients are vulnerable to Libman–Sacks endocarditis.^[15]^ Of these, approximately 11% of patients with Libman–Sacks endocarditis experience secondary ischemic stroke.^[16]^ Dislodged thrombus from Libman–Sacks endocarditis will lead to cardiogenic embolism, which becomes 1 of the main causes of stroke secondary to cerebral embolic stroke in SLE. Mohammadian et al found that antiphospholipid antibodies and early atherosclerosis can lead to the narrowing or blockage of blood vessels, serving as a contributing basis to the narrowing of blood vessels caused by atherosclerosis.^[17]^ Additionally, autoimmune antibodies, such as the antinuclear antibody (ANA), are present in patients with SLE. These antibodies can form immune complexes against vascular endothelial cells and deposit in the vessel wall, further contributing to the development of vascular inflammation by inducing endothelial cell activation. Cerebral microangiopathy and parenchymal infarction secondary to perivascular inflammation formed by a small number of mononuclear cells, as well as extensive parenchymal and vascular endothelial damage, have been found in brain tissue biopsies.^[18]^ Moreover, inflammatory changes in such vessels will be progressive, causing radiographic changes only when larger lesions involve key functional areas of the brain. Furthermore, Haas et al have shown that platelets and fibrin can create thrombi that obstruct blood vessels, resulting in secondary strokes in individuals with SLE.^[19]^ According to the available evidence, thrombosis in SLE may be associated with platelet morphological changes, immune complex aggregation on the platelet surface, and alterations in prostacyclin levels. Ruiz et al noted that antiphospholipid antibodies are present in approximately 40% of SLE patients. These antibodies induce a thrombogenic precursor state, which causes thrombosis and converts vascular endothelial cells to a pro-inflammatory phenotype.^[20]^

## 5. Diagnosis

Strokes secondary to SLE are often assumed to be strokes from other causes, suggesting difficulties in early recognition of the disease. The diagnosis of stroke secondary to SLE is an area of interest in neurology, immunology, and laboratory medicine. Brain-reactive autoantibodies, including β2GPI, are present in SLE, as demonstrated in studies of marker detection.^[21]^ Additionally, antineutrophil cytoplasmic antibody (ANCA) has been shown to play a significant role in both primary and secondary vasculitis. Moreover, a higher DEFB4 gene copy number has been associated with SLE.^[22]^ These important markers can aid in the early diagnosis of stroke secondary to SLE. Compared to serological tests, radiological imaging plays a more important role in the diagnosis of stroke secondary to SLE. HR-MRI is the only noninvasive imaging technique that visualizes and analyzes the structure of the arterial wall. Particularly in vascular inflammatory reactions, HR-MRI not only provides information on the morphological changes in different vessels but also assesses the activity of vasculitis and helps to select lesions of interest for biopsy.

## 6. Therapeutic advances

Currently, many physicians are employing intravenous thrombolytic therapy, mechanical thrombectomy, and pharmacological conservative treatment to address acute ischemic strokes secondary to SLE under secure circumstances. Rt-PA, with its high safety and bioavailability profile, is gaining recognition as a promising therapeutic intervention for stroke in patients with SLE. As illustrated in Table 2, rt-PA exhibited remarkable efficacy in the patient with NIHSS scores exceeding 3. The prognosis of patients who received intravenous thrombolysis with rt-PA showed significant improvement. Given the time-dependent nature of intravenous thrombolysis, intravenous thrombolysis for such patients should be performed with careful consideration of the time factor and active prevention of early hemorrhagic transformation.^[6]^ Mechanical thrombectomy provides a life-saving treatment option for cerebral artery embolism stemming from Libman Sacks endocarditis in certain patient populations. Conversely, conservative pharmacological treatment is utilized for other patients owing to their exceeding the venous thrombolysis time window and their unwillingness to undergo mechanical thrombectomy. Currently, the management of patients with stroke attributed to SLE relies heavily on antiplatelet drugs, antihypertensive medications, and immunosuppressive agents. In clinical trials such as CHANCE and POINT, patients with AIS who initiated aspirin within 24 to 48 hours of symptom onset experienced a 13% lower relative risk of disease recurrence. Additionally, clopidogrel-aspirin treatment reduced the risk of major ischemic events at 90 days compared with aspirin alone. However, the benefit of dual antiplatelet therapy appeared to be confined to the first 21 days after a minor ischemic stroke.^[23]^ In adults, a low-dose aspirin regimen has been shown to provide significant protection against thrombotic events in patients with lupus who are positive for antiphospholipid antibodies (aPL). Several patients receiving dipyridamole therapy for stroke demonstrated symptom improvement and did not manifest excessive platelet aggregation. In addition, the use of immunosuppressive drug therapy may become increasingly important.^[19]^ Several prospective and retrospective cohort studies have demonstrated that hydroxychloroquine (HCQ), the mainstay of SLE treatment, is associated with a reduced relative risk of arterial thrombosis overall.^[24]^ The combination of HCQ with low-dose aspirin may be beneficial as a stroke-prevention regimen, although this requires further validation in clinical trials. In our case, long-term use of HCQ by patients is a foundational medication for SLE treatment. HCQ is thought to exert a protective effect on the vascular endothelium, potentially preventing immune-inflammation within the blood vessels from reaching vasculitis proportions. Notably, HR-MRI revealed that the right thalamocortical geniculate artery (PCA-P2 segment) lacked the characteristic “circular” enhancement. Despite evidence of vasculitis in other cerebral arteries, no cerebral infarction occurred in the blood-supplying region, suggesting that stroke secondary to SLE may result from a combination of pathogenic factors. There is a lack of expert consensus and guidelines for the long-term prevention of ischemic stroke in SLE. According to the AHA/ASA organization, patients with ischemic stroke or transient ischemic attack due to symptoms caused by megakaryocytopenia-associated vasculitis should immediately commence high-dose glucocorticosteroids.^[9]^ This approach has been associated with a reduced risk of recurrent stroke.

**Table 2:** Reported cases of ischemic stroke associated with SLE.

No.	Source	Time	Age (yr) / gender	Diagnosis	NIHSS	Localized diagnosis	Treatment	Prognosis
1	Loharia et al^[3]^	2015	37-yr-old female	Acute ischemic stroke with SLE	More than 6 points	Acute middle cerebral artery (MCA) stroke	10 mg as IV bolus followed by 80 mg as IV infusion, treatment initiated at 4 h after stroke onset	Remission
2	Lemarroy et al^[4]^	2016	22-yr-old female	Acute ischemic stroke with SLE	10 points	Acute ischemic stroke involving a large territory of the right MCA	0.6 mg/kg, treatment started at 150 min after stroke onset	Remission
3	Maja Rubinić Majdak et al^[5]^	2016	40-yr-old female	Acute ischemic stroke with SLE	15 points	Acute infarction in the supply area of right MCA	rt-PA (0.9 mg/kg, treatment imitated at 90 min after symptom onset	Remission
4	Xiaodong Chen et al^[6]^	2017	45-yr-old female	Acute ischemic stroke with SLE	11 points	Occlusion of the right proximal MCA	rt-PA (0.9 mg/kg, 5.5 mg as bolus followed by 49.5 mg as infusion) was initiated 195 min after the stroke onset.	Remission
5	Katharina Stadler et al^[7]^	2015	A 48-yr-old female	Acute ischemic stroke with SLE	3 points	Left MCA occlusion	Mechanical thrombectomy	Remission

## 7. Conclusion

Early diagnosis and treatment of patients with SLE secondary to minor stroke (NIHSS score less than or equal to 3) may be challenging for clinicians. However, patients with a prior history of SLE should be adequately considered before determining a treatment plan, which can help slow the progression of SLE and reduce disability and death risk from stroke. In essence, stroke may even be 1 of the key manifestations of SLE, and SLE patients may even experience stroke at a relatively young age. Additionally, therapeutic modalities such as intravenous thrombolysis, mechanical thrombectomy, and pharmacological conservative therapy are being adequately utilized in the patient population with SLE secondary to ischemic stroke. Considering the success of this case and others reported in the literature, we believe it is worthwhile to further investigate the feasibility of intravenous thrombolysis for the treatment of ischemic stroke secondary to SLE. In essence, stroke may even be 1 of the key manifestations of SLE, and SLE patients may even experience stroke at a relatively young age. Additionally, therapeutic modalities such as intravenous thrombolysis, mechanical thrombectomy, and pharmacological conservative therapy are being adequately utilized in the patient population with SLE secondary to ischemic stroke. Considering the success of this case and others reported in the literature, we believe it is worthwhile to further investigate the feasibility of intravenous thrombolysis for the treatment of ischemic stroke secondary to SLE.

## Acknowledgments

Funding from the National Natural Science Foundation of Jilin Province is gratefully acknowledged.

## Author contributions

**Conceptualization:** Guanglin Liu, Hongjian Guan.

**Data curation:** Hongjian Guan.

**Formal analysis:** Hongjian Guan.

**Funding acquisition:** Hongjian Guan.

**Investigation:** Guanglin Liu, Yong Wang.

**Methodology:** Guanglin Liu, Hongjian Guan.

**Project administration:** Hongjian Guan.

**Software:** Yong Wang.

**Supervision:** Guanglin Liu.

**Visualization:** Yong Wang.

**Writing – original draft:** Guanglin Liu, Yong Wang.

**Writing – review & editing:** Guanglin Liu, Yong Wang.
